# Abdominal Ultrasound and Treatment of Hepatocellular Carcinoma

**DOI:** 10.3390/diagnostics11071268

**Published:** 2021-07-15

**Authors:** Kazushi Numata

**Affiliations:** Gastroenterological Center, Yokohama City University Medical Center, 4-57 Urafune-cho, Minami-ku, Yokohama 232-0024, Kanagawa, Japan; kz-numa@urahp.yokohama-cu.ac.jp; Tel.: +81-45-261-5656

Liver cancer is the sixth most common cancer and the third most common cause of cancer death, based on Global Cancer Statistics 2020 [1]. Hepatocellular carcinoma (HCC) is the most common type of primary liver cancer [2].

The liver imaging reporting and data system (LI-RADS) is a comprehensive system for standardizing liver imaging in patients at risk of developing HCC. In general, an abdominal ultrasound (US) is the initial screening modality for the detection of HCC. Contrast-enhanced computed tomography (CECT), and contrast-enhanced magnetic resonance imaging (CEMRI) are recommended as first-line diagnosis methods for HCC diagnosis because of their powerful differential diagnosis of liver neoplasms [3]. According to the development of US contrast agents and US systems, contrast-enhanced US (CEUS) is able to characterize focal liver lesions in detail. The European Federation of Societies for Ultrasound in Medicine and Biology guidelines highlight the role of CEUS as a cost-effective technique with a good safety profile for the characterization and detection of focal liver lesions [4]. The side effects of US contrast agents are usually mild and rare. There are no hepatotoxic or nephrotoxic effects, and there is no need to assess liver or kidney function prior to this imaging. When a blood pool contrast agent (SonoVue^®^, Bracco, Milan, Italy) was used for abdominal imaging as a US contrast agent, no deaths occurred among the 23,188 enrolled patients [5]. When used for characterizing focal liver lesions, a perflubutane-based microbubble contrast agent (Sonazoid^®^, GE Healthcare, Oslo, Norway) was reported to be associated with a low incidence of mild adverse effects, including tolerable myalgia (3.7%), abdominal pain (1.9%), and headache (1.9%), and had no serious adverse effects [6].

However, the American Association for the Study of Liver Diseases (AASLD) does not accept CEUS as a reliable diagnostic technique, as several studies have suggested that HCC can be hardly distinguished from some other non-HCC malignancies, such as intrahepatic cholangiocarcinoma and mixed hepatocellular and cholangiocarcinoma in CEUS, thus leading to inappropriate clinical strategies being applied [7,8].

In this Special Issue regarding “Abdominal Ultrasound and Treatment of Hepatocellular Carcinoma”, Li et al. perform a meta-analysis to evaluate the diagnostic accuracy of CEUS LI-RADS category 5 (LR5) for diagnosing HCC and CEUS LI-RADS category M (LRM) for characterizing in other non-HCC malignancy patients at risk of HCC in comparison to CT/MRI LI-RADS LR5 and LRM [9]. A pooled analysis revealed a sensitivity of 69% and a specificity of 93% for CEUS LR5 and a sensitivity of 67%, and a specificity of 93% for CT/MRI LR5 for diagnosing HCC. Their study also indicated that the pooled sensitivity and specificity for characterizing other non-HCC malignancies with respect to CEUS LRM were 84% and 90%, respectively, and that those of CT/MRI LRM were 63% and 95%. Therefore, this meta-analysis indicated that CEUS LI-RADS can be reliably used to characterize HCC and other non-HCC malignancies and may provide complementary information on liver nodules to CT/MRI LI-RADS [9].

The current version of CEUS LI-RADS (version 2017) is applicable only to pure blood pool contrast agents such as Lumason^®^ (Bracco Diagnostics, Monroe Township, NJ, USA) (as same as SonoVue^®^) and Definity^®^ (Lantheus Medical Imaging, Billerica, MA, USA), but not to combined blood pool and Kupffer cell contrast agents such as Sonazoid^®^. Unlike pure blood pool contrast agents, combined blood pool and Kupffer cell contrast agents have a post-vascular phase, which is defined as occurring about 10 min after injection. As liver lesions that have decreased or no Kupffer cells appearing as defective areas during the post-vascular phase of CEUS with Sonazoid, Sugimoto et al. defined that nodules ≤ 1 cm with arterial phase hyperenhancement, no early washout (within 60 s), and defective areas in the post-vascular phase should be classified as LR-5 [10]. Liver lesions showing early washout, defective areas in the post-vascular phase, and/or rim enhancement in the arterial phase were classified as LR-M. A total of 104 lesions in 104 patients were evaluated. The 48 (46.2%) LR-5 lesions included 45 HCCs, 2 high-flow hemangiomas, and 1 adrenal rest tumor. The positive predictive value (PPV) of LR-5 for HCC was 93.8% (95% confidence interval (CI): 82.8–98.7%). The 22 (21.2%) LR-M lesions included 16 non-HCC malignancies and 6 HCCs, including 4 poorly differentiated HCCs. The PPV of LR-M for non-HCC malignancies, including six intrahepatic cholangiocarcinomas, was 100% (95% CI: 69.8–100%). Therefore, in the modified CEUS LI-RADS for Sonazoid, LR-5 and LR-M are good predictors of HCC and non-HCC malignancies, respectively [10]. Similar to this result, we have to ensure that poorly differentiated HCC has the tendency to exhibit an early washout, as evaluated by CEUS with Sonazoid [11].

Geyer et al. evaluated 160 patients with unclear liver lesions who underwent CEUS followed by liver biopsy [12]. Comparing with histopathological results as the reference standard, CEUS with SonoVue showed a sensitivity of 94.5%, a specificity of 70.6%, a true positive rate of 87.3%, and a true negative rate of 85.7%, without the occurrence of any adverse side effects. Amongst the liver lesions, which CEUS wrongly classified as malignant, histopathology most frequently revealed fibrotic lesions (*n* = 7) and cirrhotic lesions (*n* = 3). In patients with advanced liver cirrhosis, we have to be aware of the fact that the destruction of the hepatic parenchyma may result in confluent liver fibrosis, which is known to show varying characteristics in dynamic contrast-enhanced imaging [12].

The European Federation of Societies for Ultrasound in Medicine and Biology guidelines also recognizes CEUS as a cost-effective technique with a good safety profile for monitoring tumor response after curative, loco-regional, or systemic treatment for HCC [4]. Transarterial chemoembolization (TACE) is mainly indicated for HCC patients with the Barcelona Clinic Liver Cancer (BCLC) stage B or HCC patients with BCLC stage A that have no candidate for RFA. Shiozawa et al. performed CEUS with Sonazoid within three days after TACE with drug-eluting beads (DEB-TACE) for 39 HCC lesions and investigated whether enhancement patterns can be used to predict the early therapeutic efficacy of DEB-TACE [13]. High complete response rates one month after treatment were found for lesions with no enhancement (94.1%) and peripheral ring enhancement (85.7%). The complete response rates at both 1 and 12 months after treatment were also significantly higher for lesions with no enhancement than for those with partial enhancement. CEUS immediately after DEB-TACE may allow the early assessment of therapeutic efficacy, with findings of no enhancement or peripheral ring enhancement suggesting a positive outcome [13].

Radiotherapy has excellent local control and overall survival in patients with good liver function, and it appears to be an acceptable alternative treatment option for patients who are not candidates for RFA [14,15]. Funaoka et al. investigated the use of CEUS with Sonazoid for evaluating the efficacy of radiotherapy for HCC [16]. Fifty-nine patients with 59 HCCs were evaluated retrospectively. Tumor size and tumor vascularity were evaluated using CEUS with Sonazoid before and 1, 3, 7, 10, and 13 months after radiotherapy. The median follow-up period was 44.5 months (range: 16–82 months). Compared with cases with local recurrence, the tumor size reduction and reduction in tumor vascularity (*p* < 0.001) were significantly greater in cases with no local recurrence 13 months after radiotherapy. The re-injection of Sonazoid during the post-vascular phase was an effective method for evaluating the vascularity of the target HCC itself and the surrounding liver parenchyma showing the defective area in the post-vascular phase caused by radiotherapy. CEUS with Sonazoid may be useful for evaluating radiotherapy efficacy for HCC [16].

In addition, hypervascular HCCs with an isoechoic or unclear margin on B-mode appeared as defective areas during the post-vascular phase CEUS with Sonazoid, and we could puncture them in real-time. CEUS with Sonazoid was useful for the guidance of percutaneous ablation therapy, such as the radiofrequency ablation (RFA) of HCCs not detected by conventional US [17]. Moreover, fusion imaging combining CEUS with Sonazoid and arterial-phase CECT as a reference was a useful method for evaluating the therapeutic efficacy of RFA for hypervascular HCCs detected using B-mode [18]. Fusion imaging combining CEUS with Sonazoid and the hepatobiliary phase of contrast-enhanced MRI with gadolinium ethoxybenzyl diethylenetriaminepentaacetic acid (Gd-EOB-DTPA, Primovist^®^; Bayer Schering Pharma AG, Berlin, Germany; EOB-MRI) was also useful for evaluating the therapeutic efficacy of RFA for HCCs identified as having isoechoic or unclear margins on B-mode [19].

Radiomics is a technology based on the quantitative extraction of image characteristics from radiological imaging modalities. Artificial intelligence (AI) algorithms are the principal axis of the radiomics procedure and may provide various results from large data sets beyond conventional techniques. Maruyama et al. reviewed the application of the radiomics-related diagnosis of HCC using radiological imaging (CT, MRI, and US), and discussed the current role, limitation, and future of US [20]. Investigators have shown the effect of using the US-based radiomics approach for the prediction of tumor characteristics and malignant potential posttreatment efficacy and prognosis. However, evidence has shown that the actual significance of AI-based US examinations is still far behind the effect of CT or MRI. The advantages of US are its simplicity, noninvasiveness, and real-time observation. Meanwhile, the disadvantages of US are operator and patient-dependent variations. The disadvantages of US have a great influence on each step of the workflow of radiomics, which may also be linked to the small number of US-based radiomics studies that have taken place. The major future direction of US-based radiomics may depend on how to utilize the US data, such as law data, cine clip data including multiple frames, and three-dimensional data. Moreover, comprehensive integration using a broad spectrum of laboratory data may help to improve the potential of AI-related US examination. The advantage of radiomics is operator independence, which may overcome one of the disadvantages of US in the near future [20].

The number of HCC patients experiencing metabolic dysfunction-associated fatty liver disease are significantly increasing year by year. CECT and CEMRI are generally recommended for use in obese patients. On the other hand, severely obese individuals are sometimes too big to fit in the CECT or CEMRI unit. In such cases, US may be the only modality for the screening of hepatic lesions. The further development of US systems that can detect HCCs located in deep portions of the liver and evaluate its vascularity even in cases of severely obese patients is necessary.

In closing, I believe that this Special Issue, including two review articles and four original articles, will contribute to providing a more comprehensive understanding of the diagnosis and evaluation of the therapeutic efficacy of HCC using abdominal US, especially CEUS.

## References

[1] Sung H., Ferlay J., Siegel R.L., Laversanne M., Soerjomataram I., Jemal A., Bray F. (2021). Global Cancer Statistics 2020: GLOBOCAN Estimates of Incidence and Mortality Worldwide for 36 Cancers in 185 Countries. CA Cancer J. Clin..

[2] Paradis V. (2013). Histopathology of hepatocellular carcinoma. Recent Results Cancer Res..

[3] Marrero J.A., Kulik L.M., Sirlin C.B., Zhu A.X., Finn R.S., Abecassis M.M., Roberts L.R., Heimbach J.K. (2018). Diagnosis, Staging, and Management of Hepatocellular Carcinoma: 2018 Practice Guidance by the American Association for the Study of Liver Diseases. Hepatology.

[4] Dietrich C.F., Nolsøe C.P., Barr R.G., Berzigotti A., Burns P.N., Cantisani V., Chammas M.C., Chaubal N., Choi B.I., Clevert D.A. (2020). Guidelines and Good Clinical Practice Recommendations for Contrast-Enhanced Ultrasound (CEUS) in the Liver-Update 2020 WFUMB in Cooperation with EFSUMB, AFSUMB, AIUM, and FLAUS. Ultrasound Med. Biol..

[5] Piscaglia F., Bolondi L. (2006). The safety of Sonovue in abdominal applications: Retrospective analysis of 23188 investigations. Ultrasound Med. Biol..

[6] Chou Y.H., Liang J.D., Wang S.Y., Hsu S.J., Hu J.T., Yang S.S., Wang H.K., Lee T.Y., Tiu C.M. (2019). Safety of perfluorobutane (Sonazoid) in characterizing focal liver lesions. J. Med. Ultrasound.

[7] Vilana R., Forner A., Bianchi L., García-Criado Á., Rimola J., De Lope C.R., Reig M., Ayuso C., Brú C., Bruix J. (2010). Intrahepatic peripheral cholangiocarcinoma in cirrhosis patients may display a vascular pattern similar to hepatocellular carcinoma on contrast-enhanced ultrasound. Hepatolology.

[8] Galassi M., Iavarone M., Rossi S., Bota S., Vavassori S., Rosa L., Leoni S., Venerandi L., Marinelli S., SanGiovanni A. (2013). Patterns of appearance and risk of misdiagnosis of intrahepatic cholangiocarcinoma in cirrhosis at contrast enhanced ultrasound. Liver Int..

[9] Li L., Hu Y., Han J., Li Q., Peng C., Zhou J. (2021). Clinical Application of Liver Imaging Reporting and Data System for Characterizing Liver Neoplasms: A Meta-Analysis. Diagnostics.

[10] Sugimoto K., Kakegawa T., Takahashi H., Tomita Y., Abe M., Yoshimasu Y., Takeuchi H., Kasai Y., Itoi T. (2020). Usefulness of Modified CEUS LI-RADS for the Diagnosis of Hepatocellular Carcinoma Using Sonazoid. Diagnostics.

[11] Wang F., Numata K., Nakano M., Tanabe M., Chuma M., Nihonmatsu H., Nozaki A., Ogushi K., Luo W., Ruan L. (2020). Diagnostic Value of Imaging Methods in the Histological Four Grading of Hepatocellular Carcinoma. Diagnostics.

[12] Geyer T., Clevert D.-A., Schwarz S., Reidler P., Gassenmaier S., Knösel T., Rübenthaler J., Schwarze V., Armbruster M. (2021). Diagnostic Value of CEUS Prompting Liver Biopsy: Histopathological Correlation of Hepatic Lesions with Ambiguous Imaging Characteristics. Diagnostics.

[13] Shiozawa K., Matsui T., Murakami T., Watanabe M., Maetani I. (2021). Predicting Therapeutic Efficacy of Transarterial Chemoembolization with Drug-Eluting Beads for Hepatocellular Carcinoma Using Contrast-Enhanced Ultrasound. Diagnostics.

[14] Hara K., Takeda A., Tsurugai Y., Saigusa Y., Sanuki N., Eriguchi T., Maeda S., Tanaka K., Numata K. (2019). Radiotherapy for Hepatocellular Carcinoma Results in Comparable Survival to Radiofrequency Ablation: A Propensity Score Analysis. Hepatology.

[15] Kim N., Cheng J., Jung I., Liang J.D., Shih Y.L., Huang W., Kimura T., Lee V.H.F., Zeng Z.C., Zhenggan R. (2020). Stereotactic body radiation therapy vs. radiofrequency ablation in Asian patients with hepatocellular carcinoma. J. Hepatol..

[16] Funaoka A., Numata K., Takeda A., Saigusa Y., Tsurugai Y., Nihonmatsu H., Chuma M., Fukuda H., Okada M., Nakano M. (2021). Use of Contrast-Enhanced Ultrasound with Sonazoid for Evaluating the Radiotherapy Efficacy for Hepatocellular Carcinoma. Diagnostics.

[17] Numata K., Morimoto M., Ogura T., Sugimori K., Takebayashi S., Okada M., Tanaka K. (2008). Ablation therapy guided by contrast-enhanced sonography with Sonazoid for hepatocellular carcinoma lesions not detected by conventional sonography. J. Ultrasound Med..

[18] Numata K., Fukuda H., Morimoto M., Kondo M., Nozaki A., Oshima T., Okada M., Takebayasi S., Maeda S., Tanaka K. (2012). Use of fusion imaging combining contrast-enhanced ultrasonography with a perflubutane-based contrast agent and contrast-enhanced computed tomography for the evaluation of percutaneous radiofrequency ablation of hypervascular hepatocellular carcinoma. Eur. J. Radiol..

[19] Nishigori S., Numata K., Irie K., Fukuda H., Chuma M., Maeda S. (2018). Fusion imaging with contrast-enhanced ultrasonography for evaluating the early therapeutic efficacy of radiofrequency ablation for small hypervascular hepatocellular carcinomas with iso-echoic or unclear margins on conventional ultrasonography. J. Med. Ultrasond.

[20] Maruyama H., Yamaguchi T., Nagamatsu H., Shiina S. (2021). AI-Based Radiological Imaging for HCC: Current Status and Future of Ultrasound. Diagnostics.

